# Risk factors for development of ventricular tachycardia in patients with ventricular premature contraction with a structurally normal heart

**DOI:** 10.1002/joa3.12286

**Published:** 2019-12-16

**Authors:** Yuichi Nomura, Syunji Seki, Daisuke Hazeki, Kentaro Ueno, Yuji Tanaka, Kiminori Masuda, Makoto Nishibatake, Masao Yoshinaga

**Affiliations:** ^1^ Committee on the School‐based ECG Screening Program of Kagoshima City Medical Association Kagoshima Japan; ^2^ Department of Pediatrics Kagoshima City Hospital Kagoshima Japan; ^3^ Kagoshima University Hospital Kagoshima Japan; ^4^ National Hospital Organization Kagoshima Medical Center Kagoshima Japan; ^5^ Kagoshima Seikyo Hospital Kagoshima Japan

**Keywords:** risk factors, school‐based cardiovascular screening, structurally normal heart, ventricular premature contraction, ventricular tachycardia

## Abstract

**Background:**

We examined risk factors for development of ventricular tachycardia (VT) in pediatric patients with ventricular premature contractions (VPCs) and a structurally normal heart.

**Methods:**

The subjects were 81 844 first graders and 88 244 seventh graders of Kagoshima City School‐based cardiovascular screening (SCV‐screening) between 2001 and 2015. We retrospectively reviewed the clinical data of students who were diagnosed as having VPC.

**Results:**

Ventricular premature contractions were observed in 134 first graders (0.16%) and 270 seventh graders (0.31%). On the screening electrocardiograms (ECGs), 43 patients (11%) showed bi‐/trigemini, three patients (0.7%) showed a couplet, and one patient showed VT. We obtained 166 patients' follow‐up information and evaluated 59 patients (36%) as improved, 97 patients (58%) as no change, and 10 patients (6%) as worsened (couplets, five; triplets, two; VT, three). We assumed that these worsened patients have risk factors for development of VT. Comparing the findings of SCV‐screening ECGs of risk patients with the others, a significant difference was observed only in the number of VPCs (per 10 seconds) (mean ± SD; 4.3 ± 2.6 vs 1.8 ± 1.4, *P* < .0001). A logistic regression analysis revealed that the number of VPCs was significant (*P* < .001, odds ratio; 2.01, 95% confidence intervals; 1.46‐2.93). Receiver operating characteristics analysis showed an adequate cut‐off number of three VPCs for the risk, the sensitivity was 89% and the specificity was 77%.

**Conclusions:**

Of the patients with VPC and a structurally normal heart, a few patients developed VT. Careful observation is important in patients who had three or more VPCs on SCV‐screening ECG.

## Graphical abstract

Patients with ventricular premature contraction and a structurally normal heart diagnosed through School‐based cardiovascular screening commonly showed improved or no changes in ECG during follow‐ups, but a few patients developed VT.

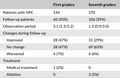

## 1 BACKGROUND

Ventricular premature contractions (VPCs) are commonly observed, not only in structural heart diseases, but also in structurally normal heart. Although the prognosis of VPC is generally considered good in a child with a structurally normal heart,^1^ a small number of patients may experience worsened arrhythmia like ventricular tachycardia (VT). The risk of VT in patients with VPC with a structurally normal heart is not fully determined, and the incidence of those VPC patients in general pediatric population, which must be a basic information concerning its risk, is not also well‐known. For adequate management of the patients with VPCs and to decrease parents' anxiety about their children, it is necessary to clarify the incidence of VPC with a structurally normal heart and to examine their risk factors for development of VT.

A nationwide school‐based cardiovascular screening (SCV‐screening) program for heart disease is implemented for all students of first, seventh, and tenth graders in Japan.^2^ This screening system is useful for identifying high risk individuals among both athletes and nonathletes.^2^, ^3^, ^4^ Using the data obtained from this program, we can assess the incidence and prognosis of VPC with a structurally normal heart in the general pediatric population.

The objective of this study was to examine the incidence and risk factors for development of VT in pediatric patients with a structurally normal heart, using VPC patients diagnosed in SCV‐screening.

## 2 METHODS

In the Kagoshima City SCV‐screening programs, students were examined at each school using 12‐lead electrocardiograms (ECGs) for 10 seconds at a paper speed of 25 mm/s. Physical examination of all students by school doctors was also performed. Parents of the students were asked to submit a questionnaire concerning their children's heart diseases. With the information from the school doctors' examination, pediatric cardiologists read ECGs and identified students requiring a second examination. Using the information from the questionnaires, pediatric cardiologists identified students who had already been diagnosed with cardiac diseases or VPCs and were currently being followed‐up by pediatric cardiologists at pediatric cardiac centers, and these patients were decided not to refer to the second examination. During the second examination for patients with VPC, physical examinations by pediatric cardiologists, chest‐radiography, and exercise‐stress ECG tests were performed. Ultrasound echocardiography was also performed, if necessary. After the second examination, patients with VPC were referred to pediatric cardiac centers for the follow‐ups.

Subjects were the first and seventh graders who were diagnosed as having VPC with a structurally normal heart for the first time in SCV‐screening between 2001 and 2015. Patients who had underlying heart disease were excluded from the study. In these VPC patients, we retrospectively reviewed the SCV‐screening ECG and clinical data from the follow‐up hospitals. We focused on the changes in presentation of VPCs during follow‐up. The changes were defined as follows: (a) improved, patients showed disappearance of VPCs; (b) no change; (c) worsened, patients with no couplets at screening ECG developed couplets, triplets, or VT, or patients with a couplet at screening ECG increased number of couplets or VT. A VT was defined as occurrence of a series of three or more consecutive VPCs.^5^ In this study, we used the term of triplet for only three consecutive VPCs. Patients of sudden cardiac death were also included in the patients with worsened change. We assumed that these worsened patients had risks of developing VT, and we examined the VPC patients diagnosed through SCV‐screening. Patients with VPCs were monitored using Holter ECG and/or an exercise ECG (Master two step test and/or Treadmill ECG). Echocardiography was performed in patients when necessary. A follow‐up period was defined as a period between SCV‐screening and the last visit of pediatric cardiac center.

This study was approved by the Ethics Committee of the Kagoshima City Hospital and it conforms to the provisions of the Declaration of Helsinki (Committee of Kagoshima City Hospital, Approval No. 2016‐30, December 14, 2016).

### 2.1 Statistical analysis

Differences in heart rate and QRS duration between worsened and nonworsened groups were examined using a Mann‐Whitney *U* test. Differences in incidences between the two groups were examined using Fisher's exact probability test. Cut‐off points were determined using receiver operating characteristic (ROC) curves to maximize the sensitivity and accuracy for discriminating patients who had risks of developing VT from the others. To examine the risk of patients, a logistic regression analysis was applied using the factors which were significantly different between patients with the risks and the others as the independent variables. All *P* values presented were two‐sided, and values of *P* < .05 were considered statistically significant. Data were processed using a statistical program SPSS 25.0 (IBM Japan, Inc).

## 3 RESULTS

### 3.1 Incidence of VPC patients

Eligible subjects for this study included 82 011 first graders (6‐7 years old) and 88 684 seventh graders (12‐13 years old) (Table 1). Students who underwent ECG in the program included 81 844 first graders (99.8%) and 88 244 seventh graders (99.5%). VPCs were observed in 134 first graders (0.16%) and 270 seventh graders (0.31%). Their incidence of each screening year varied from 0.09% to 0.28% in the first graders and from 0.21% to 0.41% in seventh graders.

**Table 1:** Prevalence of ventricular premature contraction

	First graders		Seventh graders	
	Total	VPC pts (%)	Total	VPC pts (%)
2001	5446	10 (0.18)	6303	24 (0.41)
2002	5036	14 (0.28)	5925	24 (0.41)
2003	5166	7 (0.14)	5666	15 (0.32)
2004	5275	6 (0.11)	5652	16 (0.27)
2005	5602	5 (0.09)	6213	15 (0.27)
2006	5473	10 (0.18)	6255	22 (0.35)
2007	5521	13 (0.24)	6204	21 (0.34)
2008	5544	7 (0.13)	5703	16 (0.28)
2009	5526	11 (0.20)	5825	12 (0.21)
2010	5572	8 (0.14)	5853	18 (0.31)
2011	5430	5 (0.09)	5765	15 (0.28)
2012	5259	10 (0.19)	5689	16 (0.32)
2013	5651	15 (0.27)	5737	19 (0.33)
2014	5594	6 (0.11)	5749	22 (0.40)
2015	5749	7 (0.12)	5705	15 (0.26)
	81 844	134 (0.16)	88 244	270 (0.31)

Abbreviation: VPC pts, patients with ventricular premature contraction.

### 3.2 Findings of the first screening ECG of SCV‐screening

**Figure 1:**
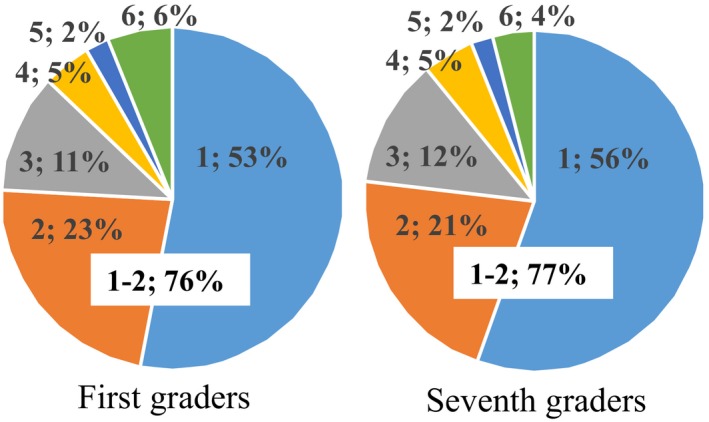
Number of ventricular premature contraction on screening electrocardiograms (ECGs). On screening ECGs, 76% of first graders and 77% of seventh graders have one or two ventricular premature contractions (VPCs). A part of the patients showed many VPCs (three or more VPCs) on screening ECGs. The ratio was 24% in first graders and 23% in seventh graders

Among patients with VPC, there were slightly more female patients in the first graders and slightly more male patients in the seventh graders (Table 2). On SCV‐screening ECGs, the number of VPCs observed was 1.8 ± 1.5 (mean ± SD) per 10 seconds in the first graders and 1.7 ± 1.5 per 10 seconds in the seventh graders. Most of the patients have one or two VPCs, those incidences were 76% in the first graders and 77% in the seventh graders (Figure 1). Bigeminy or trigeminy was observed in 14 first graders (10%) and in 29 seventh graders (10%). All patients showed monofocal VPCs, and no patients showed multifocal VPCs. A couplet was observed in one first grader and two seventh graders, and a VT was observed in one first grader. The patterns of VPCs of first graders are different from those of seventh graders (Table 3): complete right bundle branch block (CRBBB) pattern VPCs were observed in 54% of the first graders with VPC but in 35% of the seventh graders (Table 3). Most VPCs showed an inferior axis in both CRBBB and complete left bundle branch block (CLBBB) pattern.

**Table 2:** Ventricular premature contractions diagnosed in school‐based cardiovascular screening

	First graders	Seventh graders
Total students	82 011	88 684
ECG screening	81 844 (99.8%)	88 244 (99.5%)
VPC	134 (0.16%)	270 (0.31%)
Male/female	58/66	140/130
Heart rate,^a^ /min	86 ± 13	79 ± 13
Number of VPCs^a^, ^b^	1.8 ± 1.5	1.7 ± 1.5
QRS duration,^a^ s	0.11 ± 0.03	0.13 ± 0.04
Bi‐/trigeminy	14 (10%)	29 (10%)
Couplet	1 (0.7%)	2 (0.7%)
VT	1 (0.7%)	0

Abbreviations: VPC, ventricular premature contraction; VT, ventricular tachycardia.

a Data are expressed as mean ± SD.

b Number of VPCs, number of VPCs in the screening ECG for 10 s.

**Table 3:** Pattern of ventricular premature contraction

Pattern of VPC		Axis		
		Inferior	Superior	unD
First graders				
CRBBB	73 (54%)	51	12	10
CLBBB	55 (41%)	40	4	23
Undetermined	6 (4%)			
Seventh graders				
CRBBB	94 (35%)	58	24	12
CLBBB	155 (57%)	122	8	23
Undetermined	21 (8%)			

Abbreviations: CLBBB, complete left bundle branch block; CRBBB, complete right bundle branch block; unD, undetermined; VPC, ventricular premature contraction.

### 3.3 Changes in presentation of VPC during follow‐ups

Among VPC patients diagnosed in the SCV‐screening, we obtained 166 patients' follow‐up information (proportion in the VPC patients: first graders, 44%; seventh graders, 39%) (median follow‐up period: first graders, 3.1 years; seventh graders, 1.3 years). In the first graders, we evaluated 28 patients (47%) as improved, 28 patients (47%) as no change, and four patients (7%) as worsened (Table 4). During the study period, no patients with sudden cardiac death were observed, and no patients were diagnosed as having inherited arrhythmias (such as catecholaminergic polymorphic VT or long QT syndrome) or cardiomyopathy among VPC patients who were diagnosed through the SCV‐screening program. Table 5 shows the patients with worsened change in presentation of VPC. In three patients without couplets on the SCV‐screening ECG, couplets were identified in one patient (Case A) and triplets in two patients (Cases B and C) during their follow‐ups. Case D showed a couplet on the SCV‐screening ECG and further couplets were identified during follow‐up. In the seventh graders, we evaluated 31 patients (29%) as improved, 69 patients (65%) as no change, and six patients (6%) as worsened. Of three patients who did not have couplets on the SCV‐screening ECG, two patients showed couplets (Case E and F). The remaining one patient (Case G) emergently visited a pediatric cardiac center hospital with symptoms of palpitation caused by sustained VT at a 1 week after the first visit to the hospital. He underwent catheter ablation the following week (ablation site; left coronary cusp below the ostium of left main trunk) and his VPCs disappeared. In two patients who had couplets on the SCV‐screening ECG, one patient showed an increased number of couplets (Case H). The other patient (Case I) also showed an increased number of couplets and triplets. One year later, VT was identified in the treadmill exercise test. Although he was treated with propranolol and mexiletine, exercise induced VT was not alleviated. Following 2 years, two sessions of catheter ablations were carried out (ablation site in both sessions; right ventricular outflow), but the effects were insufficient. After the ablations, his condition was controlled with flecainide, with no symptoms or cardiac events during 6‐years follow‐up. Case J showed no VPC on the SCV‐screening ECG, but a school doctor pointed out her arrhythmias. She was referred to a pediatric cardiac center, and her ECG showed VPCs with couplets. During follow‐up, she exhibited sustained VT with symptoms, and 3 years later, she underwent catheter ablation successfully (ablation site; left coronary cusp below the ostium of left main trunk).

**Table 4:** Follow‐up patients with ventricular premature contractions

	First graders	Seventh graders
Patients with VPC	134	270
Follow‐up patients	60 (45%)	106 (39%)
Observation period	3.1 (1.3/5.2)	1.3 (0.9/3.0)
Changes during follow‐up		
Improved	28 (47%)	31 (29%)
No change	28 (47%)	69 (65%)
Worsened	4 (7%)	6 (6%)
Treatment		
Medical treatment	1 (2%)	0
Ablation	0	3 (3%)

Note

Observation period is expressed by median (IQR).

Abbreviation: VPC, ventricular premature contraction.

**Table 5:** Findings of the first screening ECG of school‐based cardiovascular screening in patients who showed worsened changes in presentation of VPCs

	1st/7th (sex)	HR	VPC				Bigeminy/trigeminy	Couplet/VT	Worsened changes
			n	Pattern	Axis	Duration (s)			
A	1st (F)	93	4	CLBBB	Inferior	0.12			Couplets
B	1st (M)	107	5	CLBBB	Superior	0.14			Triplets
C	1st (M)	53	3	CLBBB	Inferior	0.12	Trigeminy		Triplets
D	1st (F)	103	8	CLBBB	Inferior	0.14	Bi‐trigeminy	Couplet	Couplets
E	7th (M)	57	1	CLBBB	Inferior	0.16			Couplets
F	7th (M)	85	5	CLBBB	0°	0.16	Bigeminy		Couplets
G	7th (M)	77	7	CLBBB	Inferior	0.16	Bigeminy		VT (ablation)
H	7th (F)	93	3	CLBBB	Superior	0.12		Couplet	couplets
I	7th (M)	75	7	CLBBB	Inferior	0.17		Couplets	VT (ablation)
J	7th (F)^a^	78	7	CLBBB	Inferior	0.16		Couplets	VT (ablation)

Abbreviations: CLBBB, complete left bundle branch block; CRBBB, complete right bundle branch block; ECG, electrocardiogram; F, female; M, male; n, number of VPCs for 10 s; VPC, ventricular premature contraction; VT, ventricular tachycardia.

a School‐based cardiovascular screening ECG showed no VPCs. She was screened by the school doctor because of the arrhythmia in auscultation.

A VT observed in a first grader on the SCV‐screening ECG showed a CRBBB pattern with inferior axis. Her frequent episodes of sustained VT were confirmed by 24‐hour ambulatory ECG. Treatment with propranolol was initiated and the episode of VT gradually decreased and finally disappeared.

### 3.4 Factors of SCV‐screening ECG related with risks for development of VT

We compared the findings of SCV‐screening ECGs between the group of patients with risk for development of VT (high risk group) and the other groups (low risk group). Case J was screened by a school doctor and not by SCV‐screening ECG. We, therefore, excluded this patient from the high risk group for this analysis, and found that no differences were observed in heart rate or in the QRS duration of VPC (Table 6). The incidence of CLBBB pattern of VPC was significantly higher in the high risk group of first graders than in low risk group (*P* = .034). However, this difference was not significant in the seventh graders. Significant differences were observed in the number of VPCs (per 10 seconds), and in the incidence of bi‐/trigeminy in both first and seventh graders. The incidence of patients who had many VPCs (three VPCs or more per 10 seconds) was significantly higher in the high risk group than in the low risk group (first graders, 100% vs 29%, *P* = .011; seventh graders, 80% vs 19%, *P* = .008). The incidence of patients with a couplet was higher in high risk group. This difference was significant in the seventh graders (*P* = .002) but not in the first graders. Using the factors of number of VPCs, positive or negative in the bi‐/trigeminy pattern, or couplet as the independent factors, a logistic regression analysis revealed that the number of VPCs was an independently predictive factor for high risk patients (*P* < .001, odds ratio; 2.01, 95% confidence intervals; 1.46‐2.93). ROC analysis in the first graders revealed an adequate cut‐off number of VPCs for high risk patients (four VPCs per 10 seconds, area under curve (AUC) = 0.894) and showed that the sensitivity was 75% and the specificity was 85%. In the seventh graders, ROC analysis revealed an adequate cut‐off number of VPCs for high risk patients (three VPCs per 10 seconds, AUC = 0.833) and showed that both the sensitivity and the specificity were 80%. Cut‐off number of VPCs in all patients was three VPCs per 10 seconds (AUC = 0.863) and showed a sensitivity of 89% and specificity of 77%.

**Table 6:** Factors of school‐based cardiovascular screening ECG related with risks for development of VT

Risk for VT	First graders (59)			Seventh graders (105)		
	Low risk	High risk		Low risk	High risk	
Sex (male/female)	25/30	2/2		50/50	4/1	
Heart rate,^a^ /min	83 (57‐120)	98 (53‐107)		76 (41‐115)	98 (57‐93)	

VPC on screening ECG			*P*‐value			*P*‐value
QRS duration,^a^ ms	120 (80‐160)	130 (100‐140)	.248	140 (80‐180)	160 (120‐170)	.053
Pattern (R/L/ND)	31/21/3	0/4/0	.034	31/58/11	0/5/0	.167
Number^a^ /10 s	2 (1‐8)	4.5 (3‐8)	.006	1 (1‐6)	5 (1‐7)	.002
Bi‐/trigeminy (%)	7 (13%)	3 (75%)	.011	8 (8%)	3 (60%)	.008
Couplet (%)	0	1 (25%)	.068	0	2 (40%)	.002

Abbreviations: ECG, electrocardiogram; L, CLBBB; ND, not determined; R, CRBBB pattern; VPC, ventricular premature contraction; VT, ventricular tachycardia.

a Data are expressed as the median (range); risk for VT, risk for development of VT or increase in number of couplets.

## 4 DISCUSSION

This study showed that the incidence of VPC patients with a structurally normal heart was 0.16% in the first graders and 0.31% in the seventh graders. Among the follow‐up patients, 93% of the first graders and 94% of the seventh graders showed improved or no change in the presentation of VPCs. Worsened changes were observed in 7% of the first graders and 6% of the seventh graders. Many VPCs of SCV‐screening ECG (three or more VPCs per 10 seconds) indicated a risk for development of VT or increase in number of couplets.

The incidence of VPCs with a structurally normal heart has varied widely, primarily according to the method of detection.^1^ Twenty four‐hour ambulatory ECG is the most sensitive method. Using this method, Scott et al^6^ reported that the incidence of VPCs in 131 healthy boys aged 10‐13 years was 26%. Dickinson and Scott^7^ also reported that the incidence of VPCs in 100 healthy teenage boys was 31%. In Japan, Nagashima et al^8^ reported that the incidences of VPCs in 360 healthy children were 18%‐27%. There are two issues in these reports: (a) the numbers examined in the studies were small, and (b) all children who have VPCs are too much for pediatric cardiologists to manage. The issue regarding the small number of patients can be resolved in Japan, because a nationwide SCV‐screening program has been performed. Through the SCV‐screening, a large number of apparently normal students' ECGs are available. Using SCV‐screening ECGs, the incidence of VPCs was shown in several reports: 0.17%‐0.32% in the first graders and 0.35%‐0.50% in the seventh graders (Table 7).^9^, ^10^, ^11^, ^12^ Among these VPC patients, some patients had heart diseases. This study showed the lowest incidence among the previous reports, because we excluded patients with heart disease who had been already diagnosed before. SCV‐screening ECGs are recorded only for 10 seconds, so that students diagnosed in the SCV‐screening may have frequent VPCs (8640 or more VPCs per day, in a simple calculation). The PACES/HRS expert consensus statement showed that the management of patients with frequent VPCs, defined as more than 10% of beats in a 24‐hour, should be followed longitudinally.^13^ Considering this PACES/HRS guideline, SCV‐screened VPC patients with a structurally normal heart require further examination.

**Table 7:** Previous reports of prevalence of ventricular premature contraction in school‐based cardiovascular screening

Reference	District	Study period	First graders	Seventh graders
Hosaki^9^	Tokyo	1975‐1984	0.29% (80 368)	0.46% (50 323)
Nagano et al^10^	Yokohama	1989‐1995	0.17% (431 086)	0.35% (158 432)
Nagano et al^10^	Nagano	1989‐1995	0.32% (160 846)	0.57% (162 364)
Nagashima^11^	Nagoya	1994‐1998	0.19% (101 713)	0.35% (104 378)
Sumitomo^12^	Tokyo	1998‐2007	0.28% (492 900)	0.50% (401 487)
Present study	Kagoshima	2001‐2015	0.16% (81 844)	0.31% (88 244)

Prognosis of these VPC patients with a structurally normal heart is thought to be benign and not to be associated with sudden, unexpected death.^1^ Tsuji et al^14^ studied a prognosis of 163 children with frequent VPCs (1000 or more VPCs per day) and a structurally normal heart, and they concluded that the prognosis was generally favorable. Ehara et al^15^ examined the prognosis of 23 patients with couplets of VPC but without any underlying diseases diagnosed at SCV‐screening. These reports showed that even a more severe form of VPC showed a favorable prognosis.

Although VPC patients with a structurally normal heart mostly have a favorable prognosis, a few patients in this study developed VT. Tuji's study^14^ also reported that some patients developed new VTs and one patient died as a result of VT. The management and treatment of these patients with worsened change in presentation of VPCs remains an issue to be solved. To manage these patients adequately, the first step is to select high risk patients, patients who have a risk for development of VT or increase in number of couplets, out of many patients with improved or no change. SCV‐screening ECGs are useful, not only for screening VPC patients but also for estimating a risk for a worsened change. Patients with three or more VPCs on the SCV‐screening ECG may have a higher risk for developing VT or increasing numbers of couplets. These high risk patients should be managed with careful observation. The usefulness of SCV‐screening ECGs has been shown in this study. However, one patient who developed VT later during follow‐up was not screened by the SCV‐screening ECG. School doctors can screen patients who have arrhythmias such as VPC, but screened patients are limited in the patients with frequent VPCs. Patients screened by school doctors may be the patients who have frequent VPCs. Therefore, the SCV‐screening program itself (not only the SCV‐screening ECGs), including school doctors' physical examinations, is important for screening patients with VPC.

This study showed that CRBBB with an inferior axis was the most frequent morphology of VPCs in the first graders and that CLBBB with an inferior axis was the most frequent morphology in the seventh graders. This difference in morphology between the first and seventh graders has been previously reported.^10^ Concerning the difference between CRBBB and CLBBB morphology, Beaufort‐Krol et al examined 59 children with VPCs and reported that VPCs that originate from the left ventricle (RBBB morphology) are more likely to regress.^16^ This report may explain the difference in morphology between the first and seventh graders. Still, the mechanism is not completely understood, it may be related to a developmental process of the conduction system. All high risk patients showed the CLBBB pattern and one first grader who had VT in the first screening ECG and improved after receiving medication showed the CRBBB pattern. These findings were interesting in considering prognoses for patients with VPCs identified by SCV‐screening program.

### 4.1 Limitations

This study has some limitations: a low percentage of follow‐up students and a short follow‐up period are two of them. In Kagoshima City, any cardiac event in a student, such as sudden death, out of hospital cardiac arrest, VT, or heart failure, results in them being transferred to one of the four pediatric cardiac centers. Since the pediatric cardiologists of the four pediatric cardiac centers are members of the committee of the SCV‐screening, all students' information regarding the events are reported to the committee of the SCV‐screening and every reported patient's SCV‐screening ECG is rechecked. The committee did not receive any information of cardiac events of the VPC patients diagnosed by SCV‐screening during the study period, suggesting that no events occurred in the VPC patients who dropped out from the follow‐up in this study. The ratio of VPC patients with a favorable prognosis was larger than that shown in this study. Another limitation involved our using the ECGs at the first screening as the basement data to identify their prognosis. SCV‐screening ECGs are recorded for only 10 seconds. This short period of recording does not always reflect a patient's condition. Therefore, there might be a possibility of an underestimation of the basement data. Despite these limitations, the data shown in this study are important and useful for the pediatric cardiologists to make plans for further examination and follow‐up when VPC patients are identified through the SCV‐screening program and are referred to their hospitals. The information is also valuable for the parents of the children with VPC to ensure that they receive proper follow‐up after the initial screening.

## 5 CONCLUSIONS

The incidence of patients with VPC and a structurally normal heart in the SCV‐screening was 0.16% in the first graders and 0.31% in the seventh graders. VPC patients with a structurally normal heart diagnosed in SCV‐screening commonly showed improved or no changes in ECG during the follow‐ups, but a few patients developed VT. Careful observation is important in patients who had three or more VPCs identified on the SVC‐screening ECG.

## CONFLICT OF INTEREST

The authors declare no conflict of interests for this article.
